# Korean community hospital network for preventing and managing healthcare-associated infections: a 7-year review of activities

**DOI:** 10.1017/ash.2025.88

**Published:** 2025-09-03

**Authors:** Inhye Kwak, Myoung-jin Shin, Su young Kim, Eunsil Lee, Darae Lee, Eu Suk Kim

**Affiliations:** Seoul National University Bundang Hospital Infection Control Services, and Department of Internal Medicine, Seoul National University Bundang Hospital, Seoul National University College of Medicine, Seongnam, Republic of Korea

## Abstract

**Objectives:** Hospital networks can significantly improve healthcare-associated infection (HAI) prevention and management through quality improvement and standardization. Since 2017, the Korea Disease Control and Prevention Agency (KDCA) has coordinated a network project involving a central hospital and participating community hospitals nationwide. This paper shares our experience as one of the central hospital operating the program for seven years (2017-2023). **Methods:** Our network comprised one central tertiary-care hospital and 12 community hospitals. Activities focused on education, consultation, and quality improvement (QI). QI activities analyzed hand hygiene (HH) practices, alcohol-based hand rub consumption, and the World Health Organization’s HH Self- Assessment Framework (HHSAF) results. We provided educational resources for personal protective equipment (PPE) training, particularly during the COVID-19 pandemic. Annual workshops facilitated the sharing of specialized infection control programs from each hospital. **Results:** The project conducted 19 training sessions on topics like multidrug-resistant organism (MDRO) infection control, with 1,435 participants. We offered consultations for 41 cases (paper, phone, and 8 on-site visits) and shared consultation details through regular meetings. QI activities resulted in most hospitals maintaining a HH practice rate above 90%. All eight hospitals that consistently participated in the program saw improvements in their HHSAF scores compared to baseline. Notably, two hospitals achieved an “Advanced” level, having previously been at an “Intermediate” level. PPE training for 12,762 healthcare workers across 13 hospitals strengthened their response capabilities during COVID- 19 and reduced occupational infection risks. **Conclusions:** Frequent patient transfers and rising HAI rates highlight the limitations of individual hospitals in preventing and managing HAIs. The community hospital network establishes a government-led infection prevention response system. This model fosters enhanced infection control capabilities across network hospitals by offering technical support to resource-constrained facilities and implementing effective infection prevention initiatives that address ongoing challenges.

